# Knowledge, perceptions and attitudes of medical doctors toward elderly sexuality in Tunisia

**DOI:** 10.1192/j.eurpsy.2024.1602

**Published:** 2024-08-27

**Authors:** H. Jemli, A. Aissa, U. Ouali, R. Ezzaibi Jemli

**Affiliations:** ^1^ faculty of medicine of tunis; ^2^razi hospital, tunis, Tunisia

## Abstract

**Introduction:**

The sexual health of the elderly presents certain particularities to be taken into consideration in the doctor-patient rapport. The aim of our study was to assess physicians’ knowledge and attitudes towards sexuality in the elderly in Tunisia and to determine variables associated with the level of knowledge and the nature of attitudes in this population.

**Objectives:**

The aim of our study was to assess physicians’ knowledge and attitudes towards sexuality in the elderly in Tunisia and to determine variables associated with the level of knowledge and the nature of attitudes in this population.

**Methods:**

A descriptive and analytical study was conducted among specialists and medical residents of all specialties, practicing in Tunisia and recruited anonymously online. We included questions on socio-demographic data, medical specialty and medical training of physicians as well as a French translation of the Aging Sexual Knowlesge and Atttidues Scale (ASKAS). We determined correlations between the socio-demographic and medical training variables and the ASKAS score among participants.

**Results:**

We included 74 physicians in the study. Sixty-two percent of the doctors surveyed sometimes asked elderly patients about their sexuality (N=46) and the rest of the doctors never mentioned the subject during a medical consultation (N=28). The major obstacles reported when discussing sexuality with the elderly were: a feeling discomfort related to sexuality considered a taboo subject (77%), lack of information and skills (51%) and the duration and setting of the medical consultation considered inadequate (45%). The mean score for the knowledge subcategory of the Aging Sexual Knowledge and Atittudes Scale (ASKAS) was 68.49±5.5 and for the attitudes subcategory was 83.74±4.2. These results indicated a moderate level of knowledge and moderate to negative attitudes.

There was a significant and negative correlation between age and the ASKAS knowledge subcategory score (r= -0.75, p=0.026), as well as a significant positive correlation between the knowledge and attitudes subcategories scores and sexology training (p<0.001).

The correlation between the knowledge score and the attitudes score was significant, positive, and good (r=0.788, p<0.001): the lower the level of knowledge, the more negative the attitudes regarding elderly sexuality.

**Conclusions:**

There are several gaps in the knowledge and perceptions of Tunisian physicians regarding the sexuality of older subjects. Theoretical teaching and practical anti-ageing training for health professionals are needed.

**Disclosure of Interest:**

None Declared

